# Humanin Prevents Age-Related Cognitive Decline in Mice and is Associated with Improved Cognitive Age in Humans

**DOI:** 10.1038/s41598-018-32616-7

**Published:** 2018-09-21

**Authors:** Kelvin Yen, Junxiang Wan, Hemal H. Mehta, Brendan Miller, Amy Christensen, Morgan E. Levine, Matthew P. Salomon, Sebastian Brandhorst, Jialin Xiao, Su-Jeong Kim, Gerardo Navarrete, Daniel Campo, G. Jean Harry, Valter Longo, Christian J. Pike, Wendy J. Mack, Howard N. Hodis, Eileen M. Crimmins, Pinchas Cohen

**Affiliations:** 1 Leonard Davis School of Gerontology, University of Southern California, Los Angeles, CA USA; 2 Department of Pathology, Yale School of Medicine, New Haven, CT USA; 3 Department of Translational Molecular Medicine, John Wayne Cancer Institute at Providence Saint John’s Health Center, Santa Monica, CA USA; 4 Department of Molecular and Computational Biology, University of Southern California, Los Angeles, CA USA; 5 Neurotoxicology Group, National Toxicology Program Laboratory, National Institute of Environmental Health Sciences, Research Triangle Park, Triangle Park, NC USA; 6 Departments of Medicine and Preventive Medicine, University of Southern California Atherosclerosis Research Unit, University of Southern California, Los Angeles, CA USA

## Abstract

Advanced age is associated with a decline in cognitive function, likely caused by a combination of modifiable and non-modifiable factors such as genetics and lifestyle choices. Mounting evidence suggests that humanin and other mitochondrial derived peptides play a role in several age-related conditions including neurodegenerative disease. Here we demonstrate that humanin administration has neuroprotective effects *in vitro* in human cell culture models and is sufficient to improve cognition *in vivo* in aged mice. Furthermore, in a human cohort, using mitochondrial GWAS, we identified a specific SNP (rs2854128) in the humanin-coding region of the mitochondrial genome that is associated with a decrease in circulating humanin levels. In a large, independent cohort, consisting of a nationally-representative sample of older adults, we find that this SNP is associated with accelerated cognitive aging, supporting the concept that humanin is an important factor in cognitive aging.

## Introduction

Aging is associated with a number of biological changes including decreases in memory and cognitive function. Humanin is the first member of a new class of peptides originating from small, alternative open reading frames within the mitochondrial genome. Since it was discovered, humanin has been shown to be neuroprotective in multiple *in vitro* and animal studies^1^. The importance of the mitochondria in the etiology of Alzheimer’s disease (AD) is becoming more apparent and evidence suggests that humanin protects from various insults both in cellular models and *in vivo* models of Alzheimer’s disease^2–5^. *In vitro*, humanin protects against amyloid-beta (Aβ) toxicity as well as other familial forms of AD. *In vivo* rodent studies have shown that humanin also prevents the memory deficits caused by intracerebroventricular injection of Aß25–35 as well as having potent protective properties in the triple-transgenic AD mouse^5,6^. Thus, humanin is a neuroprotective factor whose endogenous production could influence AD progression and other neurodegenerative diseases. Because circulating humanin levels have been shown to decrease in humans as they age, humanin could also play a role in age-related cognitive decline although this has not been investigated^7^. Notably, Alzheimer mouse models carry high penetrance genetic mutations, which promote early pathology, thus preventing studies focused on cognitive decline in the old/aging brain.

Because humanin is encoded within the mitochondrial genome, mitochondrial genetics may influence the expression of humanin, which in turn may directly influence cognition during aging. In fact, many of the differences in disease incidence between haplogroups and ethnicities are in diseases that have been linked to humanin in animal models such as Alzheimer’s, diabetes, and cardiovascular disease^7–16^. Thus, in this study we examined the role of humanin in cognition and its use as a possible intervention across several experimental models and paradigms.

## Results

### Humanin protects cells and mitochondria from Aβ toxicity

**Figure 1:**
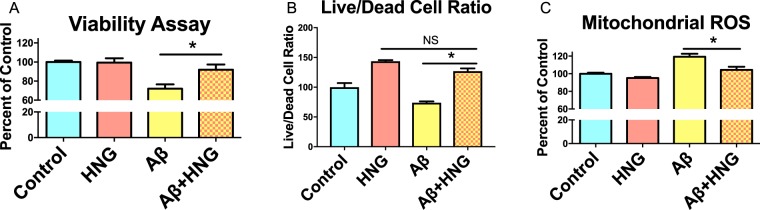
Humanin treatment reduces Aβ-induced toxicity (**A**) SH-SY5Y cells treated with Aβ (2 µM) have a decrease in viability measured by MTT and this is prevented by HNG treatment (**B**) or when measured by calcein stain (**C**) Isolated neuronal mitochondria increase ROS production upon Aβ treatment and HNG treatment prevents this induction. * indicates p < 0.05.

Humanin has been shown to be a neuroprotective agent *in vitro* against a number of toxic insults. To determine whether humanin protects against Aβ mitochondrial toxicity, we treated SH-SY5Y cells, a human neuroblastoma cell line, with soluble Aβ_1–42_ with and without humanin-S14G (HNG), a commonly used potent humanin analogue. We found that HNG protects against Aβ induced reduction of cell metabolism/viability using an MTT assay (Fig. 1A) and calcein stain (Fig. 1B). We further investigated whether this protective effect is associated with a reduction in reactive oxygen species (ROS) induced by Aβ directly in mitochondria. By isolating neuronal mitochondria from the brains of mice, we determined that Aβ causes a significant increase in ROS production and that humanin treatment can protect against this ROS increase (Fig. 1C). We verified our mitochondrial isolation technique by performing Western blots for cytoplasmic and mitochondrial markers (GAPDH and mtCOX2 respectively) (Supplementary Fig. 1). Thus, humanin may act as an *in vitro* neuroprotective agent, at least in part due to a direct effect on mitochondrial ROS production.

### Humanin decreases age-related cognitive decline in mice

**Figure 2:**
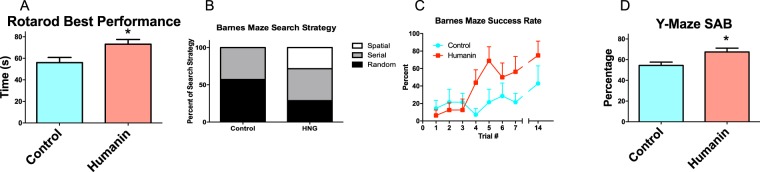
Humanin treatment reduces age-related cognitive decline. (**A**) Middle-aged mice treated with humanin perform better than control on the rotarod test after 6 months of treatment. After 10 months of treatment, treated mice had a significant improvement in (**B**) search strategy and (**C**) success rate in the Barnes maze. (**D**) The mice also performed better in the Y-maze as measured by spontaneous alternation behavior (SAB).

To examine if humanin may improve cognition in a mammalian model of aging, 18-month-old, female C57Bl/6N mice were obtained from the National Institute on Aging (NIA) and treated with HNG biweekly by IP injection at a dose of 4 mg/kg. This dose was chosen as it was shown to have physiological effects^17^. At 24-months of age (6 months of treatment) (N = 24 and 23 mice for control and HNG groups respectively), the HNG-treated group maintained their balance longer than the control group on a rotarod test (Fig. 2A). This result was not due to differences in body weight as there was not a significant difference between the two groups of mice selected for this test (data not shown). Four months later, the trend was the same but the difference between the two groups was smaller and became non-significant. On the other hand, at 28-months a subset of the mice (N = 7 or 8 for control and HNG treated groups respectively) were given a Barnes maze test; HNG-treated mice improved their search strategy (Fig. 2B) and success rate (Fig. 2C) compared to control. An additional Y-maze test was administered to another subset of mice at 28 months of age (N = 10/group); HNG-treated mice had improved spontaneous alternation behavior compared to control (Fig. 2D). Together, this suggests that the HNG treatment improves the cognitive ability of old mice.

**Figure 3:**
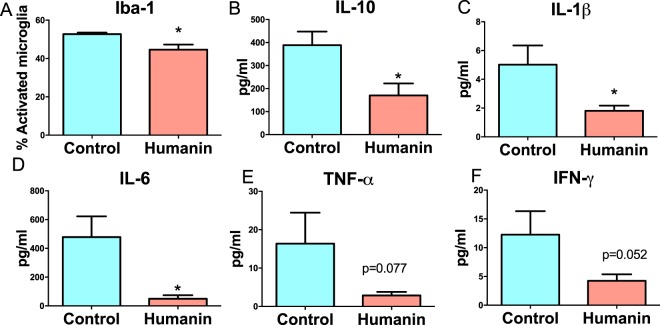
Humanin treatment reduces inflammation in mice. Mice treated with humanin analogue, HNG, have a decrease in (**A**) activated microglia detected by Iba-1 staining. They also have a general decrease in circulating inflammatory markers 3B-3F. * indicates p < 0.05.

To examine if an increase in neurogenesis could explain the improvement in cognitive ability, we examined the dentate gyrus of the mice for neurogenesis. At approximately 32 months of age, the remaining mice (n = 6 for control and 8 for HNG groups) were administered BrdU at 50 mg/kg for 8 days and then sacrificed, and plasma and brain were collected for further analysis. Hippocampal neurogenesis was examined by staining for both BrdU and doublecortin, which identifies newborn neurons. There was no significant difference in the number of BrdU or doublecortin positive cells between the two groups (Supplementary Fig. 2A and B). Because of the association between inflammation, aging, and memory decline, we examined if a reduction in pro-inflammatory, activated glia could explain the memory improvements in the HNG-treated mice^18^. We stained and morphologically analyzed the IBA1/AIF-1 positive microglia and found a significant decrease in the number of microglia exhibiting an activated phenotype in the HNG group (Fig. 3A). Further, it has been proposed that microglia change as a function of aging and that such change may reflect a diminished capability for the cells to perform their normal function^19,20^. Indeed, prior work^19–26^ suggested that some age-related morphological changes in microglia were reflective of dystrophy and senescence. A careful morphological analysis did not demonstrate any overt evidence of a dramatic shift in dystrophic microglia morphology in the HNG-dosed mice (Supplementary Fig. 3). We further examined circulating inflammatory markers using MesoScale Discovery (MSD) technology and found that other markers of inflammation were decreased in humanin compared to control (Fig. 3B–F). Together, this suggests that HNG treatment decreases both microglial activation and systemic inflammation, outcomes that could explain the observed improvements in memory and cognition.

### A SNP predicts circulating humanin levels and interacts with ancestry

**Figure 4:**
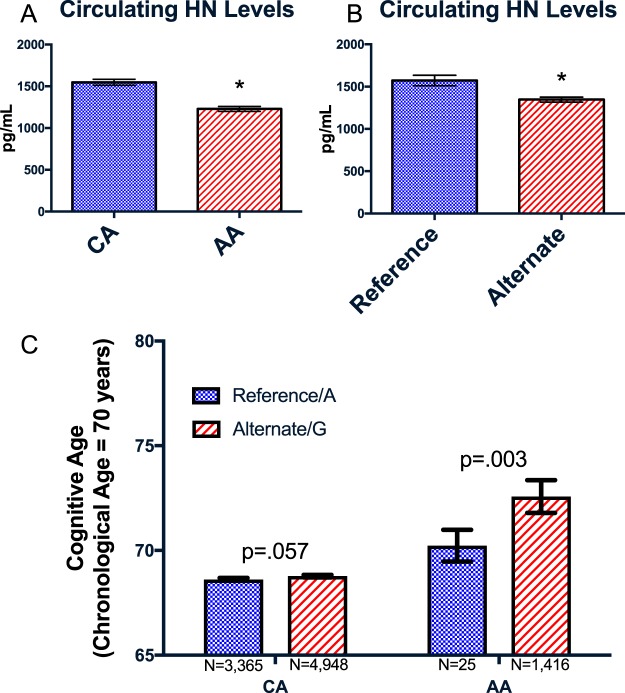
Humanin levels and cognitive age is associated with SNP rs2854128. (**A**) African-Americans have lower circulating humanin levels than Caucasian-Americans. (**B**) Individuals with the mitochondrial SNP rs2854128 have a significant decrease in circulating humanin levels. (**C**) RS2854128 is associated with an increased cognitive age that is more pronounced in African-Americans. *Signifies p < 0.05.

Having found that humanin could affect neurocognition in mice, we hypothesized that differences in circulating humanin levels could partially account for the known ethnic disparities in neurocognitive diseases between Caucasian Americans (CA) and African Americans (AA). We used samples from the B-Vitamin Atherosclerosis Intervention Trial (BVAIT) study, from which we selected 71 AA and 75 CA participants who were matched by gender, age, and randomized treatment (Table 1)^27^. This group was made up of male and female patients at least 40 years old with no signs or symptoms of cardiovascular disease. We found that the mean circulating humanin level in AA is nearly 20% lower compared to CA, p < 0.05 (Fig. 4A).

**Table 1:** Demographic characteristics and humanin levels in African American (AA) and Caucasian Americans (CA) subjects in the BVAIT study. Participants were individually matched on treatment assignment, gender and age.

Variable	CA (n = 75)	AA (n = 71)	p-value
Age	58.5 (9.6)	58.4 (10.0)	0.99
Gender			1.00
Male	39 (52%)	36 (52%)	
Female	36 (48%)	35 (48%)	
Humanin (pg/mL)	1491.0 (267.3)	1211.9 (222.7)	<0.0001

Numbers in the table are mean (SD) or n (%). P-values shown are from independent t-tests or chi-square tests.

Because humanin is encoded within the mitochondrial genome, we next examined the mitochondrial DNA (mtDNA) for SNPs that could explain the differences between circulating humanin levels in AA and CA. We sequenced the entire mitochondrial genome of all samples to determine if any SNPs correlated with humanin levels. Exploring SNPs near the humanin open reading frame (ORF), we focused on rs2854128, which is at the very end of the humanin ORF (but does not result in a change in the humanin AA sequence) and found that it was associated with a 14% decrease in circulating humanin levels (Fig. 4B) p < 0.05. In a multivariate analysis, both self-reported race and the presence of the rs2854128 SNP independently and additively contributed to predicting humanin levels.

### A humanin SNP predicts cognitive decline in humans

We subsequently examined the effect of this SNP in a separate cohort of participants from the Health and Retirement Study^28^, a longitudinal study of approximately 20,000 individuals over the age of 50 in the United States of which over 12,500 individuals consented to DNA analysis. Using this dataset, we confirmed that the ancestral/reference allele is found in 42% of CA and in much lower percentages of AA or Hispanic populations (Table 2). Looking at cognitive phenotypes hypothesized to be related to humanin action, we found that the alternative allele was associated with an increase in cognitive age at a given chronological age (p < 0.05 for the entire population), after adjusting for sex, education, and race. We found a significant interaction between race and the rs2854128 SNP (P = 0.005), such that rs2854128 had an amplified effect on cognitive aging among AA, relative to CA. Subsequent stratified analysis showed that AA with the alternate allele had cognitive ages that were about two years older than AA with the reference allele, whereas there was only a marginal increase of about 0.2 years in cognitive age among CA with the alternate compared to the reference allele (Fig. 4C).

**Table 2:** Percent Distribution of SNP rs2854128 in HRS MitoG2706A/rs2854128.

	NH White	NH Black	Hispanic	Other
G	57.6	98.3	94.0	86.9
A	42.4	1.7	6.0	13.1

## Discussion

In this study we show that humanin has neuroprotective effects both *in vitro* and *in vivo*. We further show that humanin administration is sufficient to prevent some of the normal behavioral and cognitive deficits that occur with age in common laboratory mice. This suggests that the decline in humanin seen with increasing age^7^ may be one of the reasons for the age-related decline in cognition and related physiological parameters. Separately, we have identified plasma humanin levels as a novel mitochondrial biomarker that is reduced in African Americans, further linking the mitochondria and mitochondrial peptides to health disparities. Plasma humanin has also been shown to be low in endothelial dysfunction^15,29^, and to be correlated with longevity and GH levels^30^. Of note, we recently showed that a second mitochondrial peptide, SHLP2, is also lower in African Americans compared to Caucasian Americans, and reduced levels predict the risk of prostate cancer^31^.

Here we also demonstrate for the first time a mitochondrial SNP that correlates with circulating mitochondrial-derived peptide (MDP) levels. The prevalence of this SNP differs by race/ethnic group and could explain some ethnic-specific disease differences. Because of the beneficial effects of humanin, a decrease in circulating levels could lead to an increase in several different diseases of aging, particularly in dementia. We find that this SNP does correlate with advanced cognitive age, although we recognize that these studies in human populations are correlative. Interestingly, this SNP has been separately associated with cardiovascular disease and cholesterol levels, both of which humanin has also been implicated in^32^. Interestingly, this SNP is found within the coding region of the humanin gene and is a synonymous mutation that alters the stop codon of humanin from TAA(Ochre) to TAG(Amber). How this effects circulating humanin levels is unknown, but could involve differences in termination efficiency as it has been shown that TAA is a more efficient termination sequence than TAG^33,34^. Alternatively, it could also affect the stability of the humanin mRNA or the binding efficiency of the mRNA to mRNA binding proteins.

Previous studies demonstrated that humanin prevents many of the cognitive deficits in mouse models of AD, where both intranasal administration as well as injections have equally beneficial effects. Because of humanin’s multiple effects, the exact mechanism of benefit is still unknown but could involve known humanin-related actions such as its anti-apoptotic effects, anti-amyloid oligomerization properties, or anti-inflammatory effects *(17*, *24–28)*. On a molecular level, humanin is also known to activate the trimeric IL6/CNTFR/WSX-1 receptor and FPRL receptor and lead to activation of the AKT and ERK pathways^35–38^. Other cognitive diseases have also been examined in relation to humanin with several possible mechanisms proposed^39–41^. For the first time, we find that humanin can also improve normal, age-related cognitive decline. Furthermore, our data implicate the increase in inflammation with age as a possible mediator of this cognitive decline. Inflammation during aging, sometimes called inflammaging^42^, has been suggested as being a primary cause of aging, and our findings support this idea. Interestingly, many of the effects of humanin mimic the response to dietary interventions^43–45^.

Neurodegenerative diseases such as AD and other diseases disproportionately affect certain populations and this likely has both biological and socioeconomic causes. We have now discovered a possible underlying biological factor contributing to this health disparity, as we find that there is a significant difference between the circulating levels of humanin between African Americans and Caucasian Americans. Beyond cognitive aging, because of humanin’s role in other diseases including cardiovascular disease and diabetes, circulating levels of humanin could also contribute to additional diseases that disproportionately affect African Americans. The mitochondria have been implicated in health disparities because of their central importance in many diseases and the uniparental inheritance of the genome^46^. The role of humanin in health disparities is a novel concept that further implicates the mitochondria in the biological basis of health disparities.

Many rare diseases have been shown to be directly caused by mutations of the mitochondrial genome and even more have been associated with apparently synonymous mutations. Mutations and deletions of the mitochondria genome have also been implicated in aging, and mitochondrial dysfunction is found in a vast number of diseases. In fact, a different MDP, MOTS-c, has a SNP that has been associated with longevity^47^ and its levels are reduced in endothelial dysfunction^48^. Our study is the first to find an association between a mitochondrial SNP and circulating MDP levels. This connection has even more consequences suggesting that SNPs in MDPs could be potentially relevant to additional common diseases and disease risk.

## Methods

### Cell culture assays

#### MTT

MTT assays were used to measure SH-SY5Y cell proliferation. The neuroblastoma cells were seeded at 3 × 10^4^ per well in 96-well plates in DMEM/F12 (Thermo Fisher Scientific) supplemented with 10% fetal bovine serum (Omega Scientific) until 85% confluent. Cells were treated with 100 uM of freshly reconstituted humanin in Milli-Q water (GenScript) with and without *Aβ*_*1-42*_ (CPC Scientific), prepared as previously described^1^, for 24 hours. To prevent humanin-serum interference and to support cell viability for the long 24-hour treatment duration, treatments were in DMEM/12 media supplemented with N2 (Thermo Fisher Scientific). MTT (Sigma-Aldrich) reagent (5 mg/ml) was added to each well after treatments for four hours and lysed before absorbance values were read using the SpectrMax M3 microplate reader.

#### Cell Cytotoxicity Assay

A two-color fluorescence cell viability assay (LIVE/DEAD Viability/Cytotoxicity Kit; Invitrogen (cat. L3224) was used to distinguish live cells from dead cells after humanin and *Aβ*_*1-42*_ treatment. The ratio of live to dead cells can be quantified since live cells retain the Calcein AM dye and dead cells with damaged membranes permit entry of the ethidium homodimer dye. SH-SY5Y cells were seeded at 3 × 10^4^ per well in 96-well plates in DMEM/F12 (Thermo Fisher Scientific) supplemented with 10% fetal bovine serum (Omega Scientific) until 85% confluent. Cells were treated with 100 uM of freshly reconstituted humanin in Milli-Q water (GenScript) with and without *Aβ*_*1-42*_ (CPC Scientific), prepared as previously described^1^, for 24 hours. To prevent humanin-serum interference and to support cell viability for the long 24-hour treatment duration, treatments were in DMEM/12 media supplemented with N2 (Thermo Fisher Scientific). Cytotoxicity assays were performed immediately after the 24-hour incubations according to the provided protocol.

#### Live Mitochondria Isolation

Cortical brain mitochondria were isolated from male 16-week old C57BL/6 mice. Brains were removed and rinsed in ice cold buffer containing 210 mM mannitol, 70 mM sucrose, 20 mM HEPES, 2 mM EGTA, and 0.2% (w/v) fatty acid free bovine serum albumin, pH 7.4 (ME Buffer). Cortical tissue was extracted, minced, and immediately homogenized in MEB with 5-10 Teflon pestle strokes. Homogenates were centrifuged at 1000 × *g* for 5 minutes (4 °C) to remove tissue debris. Supernatants were transferred to a clean tube and centrifuged at 3500 × *g* for 10 minutes (4 °C). Centrifugations were repeated twice. The mitochondrial pellet was re-suspended in a buffer containing 220 mM mannitol, 8 mM sucrose, 10 mM KH_2_PO_4_, 5 mM MgCl_2_, 2 mM HEPES, 1 mM EGTA, and 0.2% (w/v) fatty acid free bovine serum albumin, 10 mM succinate, and 2 uM rotenone, pH 7.2 (MAS Buffer) to a final concentration of 400 ug/ml per well^49^. To confirm the ability of our protocol to isolate mitochondria, we repeated the procedure using Western blotting and probed for mtCOX2 (SC-23984 Santa Cruz Biotechnology) and GAPDH (#7074 Cell Signaling).

#### Incubation of Live Mitochondria with HNG and *Aβ*_*1-42*_

Cortical mitochondria suspensions in MAS Buffer (250 mg/ml) were plated in a 96-well plate. To prime the mitochondria, 10 uM of HNG was administered to wells and incubated at 37 °C 15 minutes. HNG is a potent analogue of humanin that contains a glycine substitution for serine at the 14^th^ amino acid. HNG was dissolved in Milli-Q water immediately prior to mitochondria administration. Following HNG exposure, 3uM or 30 uM of *A*β_1-42_ (CPC Scientific), prepared as previously described^1^, was dispensed into wells and incubated at 37 °C for 45 minutes.

#### Live Mitochondrial-derived H_2_0_2_ Detection by Amplex Red

Mitochondria readily produce superoxide in the mitochondrial matrix, which is dismutated to H_2_0_2_ by superoxide dismutase 2. H_2_0_2_ quickly diffuses across the mitochondrial membrane to the medium and can be detected using Amplex Red (10-acetyl-3,7-dihydroxyphenoxazine, Thermo Fisher Scientific). In the presence of horseradish peroxidase (HRP), Amplex Red reacts with H_2_0_2_ and is converted to resorufin, which emits light at 590 nm when excited at 530 nm. Both Amplex Red (50 uM) and HRP (0.1 U/ml) were added to MAS Buffer during HNG and *A*β_1-42_ incubations. Fluorescence emission was detected using a SpectrMax M3 microplate reader.

### Longitudinal Study of Humanin Administration in Healthy Mice

We conducted a 14-month study of 100 female, C57BL/6N mice obtained from the NIA aged mouse colony starting at 18 months of age. The mice were given twice-weekly injections of HNG at 4-mg/kg/BW or water/vehicle IP. This dose was chosen after protective effects were recently demonstrated by a Swedish group that administered humanin in twice-weekly treatments. Survival was monitored daily and body weight and daily food intake measured weekly^50^. All experiments with mice were performed in accordance with the appropriate guidelines and regulations and approved by the University of Southern California Institutional Animal Care and Use Committee (IACUC) under protocol #20787.

### Mouse cognitive tests

#### Accelerating Rotarod

This procedure was the same as described previously^43^. At 24 months of age, the mice were placed on the rod rotating at an initial speed of 4 rpm. The rotation of the rotarod was gradually increased to 40 rpm over a 5 minute session. The time at which they fall was recorded and analyzed.

Y-maze: A Y-maze test was performed when the mice were 28 months of age. The procedure was the same as described previously^43^. In short, mice were placed in one arm of the maze and allowed to freely explore for 8 minutes. An entry into an arm was recorded only if both forepaws and hindpaws fully enter the arm. Quantification of spontaneous alternation behavior was calculated as the proportion of alternations, defined as an arm choice different from her previous two choices, to the total number of alternations.

Barnes Maze: Barnes maze tests were performed as previously described^43^. 28 month old Mice were placed in a start chamber in the middle of the maze and allowed to habituate for 30 seconds. The start chamber was then removed and the mouse was allowed to explore the maze and the time until the mouse finds the escape box was recorded. A maximum of 2 minutes was allowed for each trial.

Strategy Assessment: A random strategy was characterized by localized searches to holes separated by crosses through the maze center. A serial search strategy was characterized by a systematic search of consecutive holes (every hole or every other hole) with no maze center crosses. A spatial search strategy consisted of navigating directly to the escape hole with both error and deviation scores less or equal to 3.

### BrdU and Doublecortin Staining

At approximately 32 months of age, mice were injected with BrdU for 8 days before sacrifice (50 mg/kg). Fixed hemibrains were sectioned coronally on a vibratome at 40 μm and then the brains were processed for BrdU and doublecortin (DCX) staining. Briefly, for BrdU staining sections were pretreated with 2N HCl at 37 °C, washed, and incubated overnight in primary antibody against BrdU (1:500; Abd Serotec). For DCX staining, sections were washed and incubated overnight in primary antibody against DCX (1:2500; Santa Cruz). Sections were then washed and incubated with biotinylated anti-rat secondary antibody for BrdU or anti-goat secondary antibody for DCX (Vector) for 1 hour and processed for diaminobenzidene staining using Vectastain ABC Elite kit (Vector). Stained sections were air-dried overnight, dehydrated, then coverslipped with Krystalon (EMD Millipore). Counting was done in the dentate gyrus (all layers) and quantified per unit area (mm^2^) for BrdU and per section for DCX.

### Iba-1 staining and microglial quantification

Sections for microglia staining were processed as above without the HCl and were incubated overnight in primary antibody against Iba-1 (1:2000; Wako) and biotinylated anti-rabbit secondary antibody (Vector). For microglia morphological analyses, brain sections containing the hippocampus (bregma −1.95 to −2.18) (wt n = 6; n = 7), defined regions of interest (ROI) were identified for the CA1 and CA3 regions and the morphology of Iba-1+ microglia evaluated. Brain sections were scanned under 40x magnification (Aperio ScanScope T2 scanner, Aperio Technologies, Inc., Vista, CA) and viewed using Aperio ImageScope v.6.25.0.1117. Images were assigned random numbers and blinded for evaluation. Activation state of microglia was based on morphological analysis of Iba-1 staining in a manner consistent with prior reports^51,52^. The number of Iba-1 immunoreactive cells in the hippocampus was estimated by two-dimensional cell counts using random-sampling based on the optical dissector technique. Briefly, an Olympus BX50 microscope equipped with a motorized stage and computer-guided CASTGrid software (Olympus) was used for unbiased sampling. In sections containing well-defined CA1-CA3 subregions of hippocampus, the hippocampus (excluding the dentate gyrus) was outlined, and high magnification microscopic fields were randomly sampled. Within each field, cells within a counting frame (3000 μm^2^) was used for analysis. Microglia were classified as either type 1, (many thin, ramified processes), type 2 (short, thick processes and a rod-shaped cell body), or type 3 (no or few short non-ramified processes or many filapodial processes) cells. Type 2 and 3 microglia were considered activated.

### Proinflammatory plasma biomarkers measurement

Proinflammantory biomarkers including IL-6, IL-8, IL-10, TNF-α and INF-γ were measured by a custom metabokine 8-plex kit provided by Meso Scale Discovery (Catalog#: N051A-1, Rockville, Maryland). Briefly, 25ul of standards and samples were added and incubated for 2 hours on the MSD plate. After 3 washes, 25ul of detection antibody was added into each well and incubated for 1.5 hours. After the final wash, read buffer was added and the plate was analyzed on the Quick plex SQ120.

### Human studies

Using samples from the B-Vitamin Atherosclerosis Intervention Trial (BVAIT) approved by the University of Southern California Institutional Review Board^27^, we selected 75 AA and 75 CA participants that were matched by randomized treatment assignment (B-vitamins or placebo), gender and age. Written informed consent was obtained from all subjects before their inclusion in the study and all methods were performed in accordance with the relevant guidelines and regulations. We then measured plasma humanin levels by ELISA in these patients as previously described^53^.

### DNA extraction and next generation sequencing

DNA was extracted from buffy coats collected from blood samples of the 150 BVAIT participants using the DNeasy blood and tissue kit (Qiagen) according to the manufacturer’s directions. In short, the buffy coats were lysed using proteinase K and buffer added to optimize the DNA binding conditions. These lysates were pipetted into a column that binds the DNA using silica-membrane technology, washed, and then the DNA was eluted in water. The mitochondrial DNA was minimally amplified by PCR using two different, overlapping sets of primers using TaKaRa LA TaqDNA Polymerase. The PCR products were run on a gel to check for correct amplification and then excised and purified using the QIAquick gel extraction kit (Qiagen). Illumina sequencing libraries were prepared in a 96-well plate format according to Dunham & Friesen (2013) with the exception that DNA was sheared with dsDNA Shearase Plus (Zymo: Irvine, CA, USA) and cleaned using Agencourt AMPure XP beads (Beckman- Coulter: Indianapolis, IN, USA). Fragment size selection was also carried out using beads instead of gel electrophoresis. Libraries were quantified using the Qubit 2·0 Fluorometer (Thermo Fisher Scientific Inc.), and the fragment size distribution was determined with an Agilent Bioanalyzer 2100. The libraries were then pooled equimolarly, and the final pool was quantified via qPCR using the Kapa Biosystems Library Quantification Kit, according to manufacturer’s instructions. The pool was then sequenced in a Rapid ver. 1 Flowcell, in an Illumina HiSeq 2500 platform. Final file formatting, demultiplexing and fastq generation was carried out using CASAVA v.1·8. Sequencing reads were further sorted by 4 bp barcode using the Novobarcode application within Novocraft (www.novocraft.com). The libraries preparation, pooling, quality control, sequencing and initial file processing were all performed at the UPC Genome Core (University of Southern California, Los Angeles, CA, USA).

### SNP analysis

For each sample, we first trimmed the raw sequencing reads by quality using the SolexaQA package (ver. 1·12)^54^. All bases with a quality score of less than Q = 13 were trimmed and all reads that were less than 25 bp after trimming were removed from further analysis. We then mapped the quality-trimmed reads to the revised Cambridge reference sequence of the human mitochondrial genome (NC_012920·1) using the BWA aligner (ver. 0·7·5a)^55^. Following mapping, local realignment around insertion/deletions was performed using the IndelRealigner tool as part of the GATK package (ver. 2·6–5)^56^. After mapping and realignment, we applied the GATK UnifiedGenotyper (ver. 2·6–5) to all samples simultaneously to identify single nucleotide polymorphisms (SNPs) in our sample set (we identified 791 SNPs in the mitochondria). Having identified various SNPs in our sequencing data, the next step of analysis was to correlate the SNPs to humanin levels. In particular, we focused on SNPs contained within the small open reading frame of the humanin peptide within the 16S gene of the mitochondrial genome.

The SNP was then identified in the HRS and analyzed to determine whether the SNP was correlated with humanin-related phenotypes.

### Cognitive scores

The humanin genotype was extracted from bed, bim, and fam files and merged with cognitive scores of all individuals between 1998 and 2012. Humanin genotype was identified for 15,620 individuals and was regressed against the cognitive state score for self-respondents in 2012. Scores under 7, between 7–12, and over 12 delineate demented, cognitively impaired, and cognitively healthy status, respectively. Age, sex, race, and the previous year’s cognitive score was used in the lag-dependent variable regression model on 10,158 respondents. The beta coefficient for the humanin SNP on cognitive score was 0.195 (p = 0.006, 95% CI: 0.056–0.333).

### Cognitive aging

Cognitive age measures were based on data from wave three (1996) through wave ten (2010) of the Health and Retirement Study, using four measures of cognitive functioning—delayed recall, immediate recall, serial 7 s, and backwards counting. A cognitive age was calculated for each participant at each wave based on an algorithm proposed by Klemera & Doubal^57^ that in prior work has been used for calculating biological ages from biomarker data^58,59^. This method has been validated using both real and simulated data. Cognitive age estimates combine information from equations of chronological age regressed on each of the four cognitive functioning markers. The equation for calculating cognitive age is:

$${\rm{CogAge}}=\frac{{\sum }_{j=1}^{m}({x}_{ji}-{q}_{j})\frac{{k}_{j}}{{s}_{j}^{2}}+\frac{C{A}_{i}}{{s}_{BA}^{2}}}{{\sum }_{j=1}^{m}{(\frac{{k}_{j}}{{s}_{j}})}^{2}+\frac{1}{{s}_{BA}^{2}}}$$

where, *k*_*j*_ and *q*_*j*_ are the slope and intercept, respectively, for chronological age on each cognitive measure, *x*_*ji*_ is each the value of the cognitive measure participant *i*, *s*_*j*_ is the root mean squared error of chronological age regressed on the cognitive measure, and *CA*_*i*_ is chronological age for participant *i*. Additionally, S^2^_BA_ designates the standard deviation for the difference in cognitive age and chronological age. It is estimated by taking into account the variability in the first half of the equation, the mean variance of the cognitive measures that is explained by chronological age, and the range of chronological age, as explained in Klemera and Doubal^57^. Overall, the mean cognitive age of a population should equal the mean chronological age of the population^57^.

## Electronic supplementary material

Supplemental Figure Legends
