# Supplementary material for: Humanin Prevents Age-Related Cognitive Decline in Mice and is Associated with Improved Cognitive Age in Humans
Source: Sci Rep. 2018 Sep 21;8:14212. doi: 10.1038/s41598-018-32616-7 (PMC6154958; doi:10.1038/s41598-018-32616-7)
Supplement: Supplementary file 1 — Supplemental Figure Legends [file 41598_2018_32616_MOESM1_ESM.pdf]

Humanin Prevents Age-Related Cognitive Decline in Mice and is Associated with Improved Cognitive Age in Humans

Kelvin Yen[1], Junxiang Wan[1], Hemal H. Mehta[1], Brendan Miller[1], Amy Christensen[1], Morgan E. Levine[2], Matthew P. Salomon[3], Sebastian Brandhorst[1], Jialin Xiao[1], Su-Jeong Kim[1], Gerardo Navarrete[1], Daniel Campo[4], G. Jean Harry[5], Valter Longo[1], Christian J. Pike[1], Wendy J. Mack[6], Howard N. Hodis[6], Eileen M. Crimmins[1], Pinchas Cohen[1]*

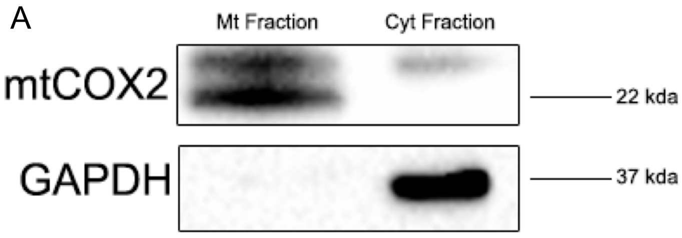

Supplemental Figure 1

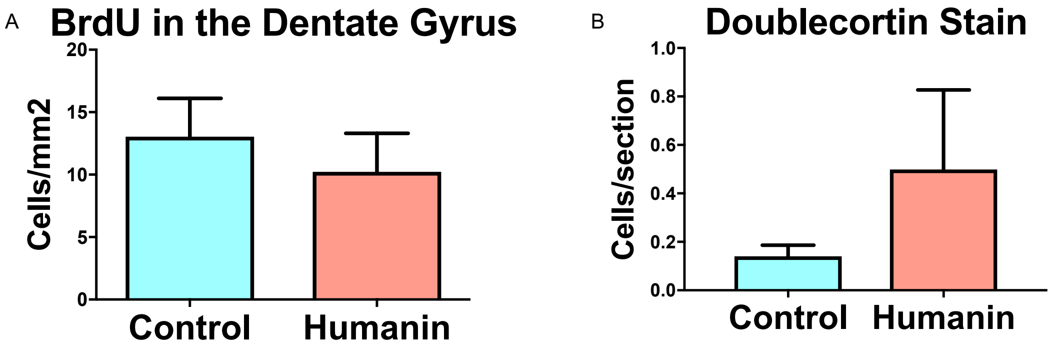

Supplemental Figure 2

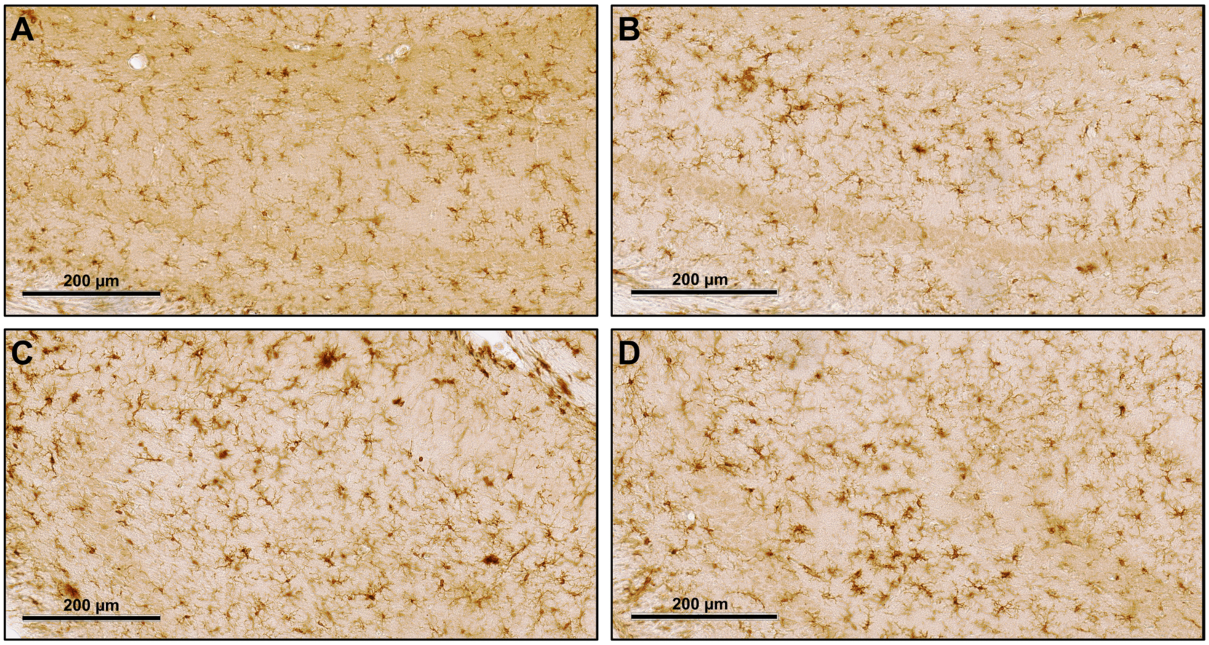

Supplemental Figure 3

Supplementary Figure Legends

Supplemental Figure 1. Verification of the purity of the cortical neuron mitochondrial protocol. Mitochondria were isolated from cortical neurons and the mitochondrial and cytoplasmic fractions were probed for a mitochondrial specific protein (mtCOX2) and cytoplasmic protein (GAPDH).

Supplemental Figure 2. There is no increase in neurogenesis in humanin treated mice. Staining for BrdU (A) and Doublecortin (B) both suggest that neurogenesis is not a cause of the increased cognitive ability of humanin treated mice.

Supplemental Figure 3. Humanin treatment does not dramatically alter microglia morphology. (A) In the aged control mice, microglia within the CA1 region showed a predominance of elongated processes with less dense process ramification that would normally be observed in young mice (Harry and Kraft, 2012). (B) In the humanin dosed mice (HNG), microglia with a similar morphology were observed however, microglia retaining a more ramified morphology were also present. The proportional distribution of elongated versus ramified morphologies appeared to be individual mouse dependent. Within the CA3 pyramidal cell region, Iba-1+ microglia within the area of mossy fiber boutons, a range of microglia morphologies could be observed in both (C) control and (D) HNG mice. These ranged from cells showing dense immunoreactivity within the cell body and shortened dense processes, cells with distinct ramification of processes, to cells showing more elongated processes. Scale bar = 200 µm.
